# Mannan Binding Lectin (MBL) genotypes coding for high MBL serum levels are associated with rheumatoid factor negative rheumatoid arthritis in never smokers

**DOI:** 10.1186/ar3321

**Published:** 2011-04-15

**Authors:** Saedis Saevarsdottir, Bo Ding, Kristjan Steinsson, Gerdur Grondal, Helgi Valdimarsson, Lars Alfredsson, Lars Klareskog, Leonid Padyukov

**Affiliations:** 1 Rheumatology Unit, Department of Medicine, Karolinska University Hospital Solna, D2:01, 17176 Stockholm, Sweden; 2 Institute of Environmental Medicine, Karolinska Institutet, Nobels väg 13, 17177 Stockholm, Sweden; 3 Department of Immunology, Landspitali University Hospital, Hringbraut (Building 14 at Eiriksgata), 101 Reykjavik, Iceland; 4 Center for Rheumatology Research, Landspitali University Hospital, Hringbraut (Building 14 at Eiriksgata), 101 Reykjavik, Iceland

## Abstract

**Introduction:**

Previous studies have provided inconsistent results on whether variants in the *MBL2 *gene, coding for the complement-activating mannan-binding lectin (MBL) protein, associate with rheumatoid arthritis (RA). We re-evaluated this in context of the main environmental and genetic risk factors (smoking, HLA-DRB1 'shared epitope' (SE), PTPN22*620W), which predispose to rheumatoid factor (RF) and/or anti-citrullinated-protein antibody (ACPA)-positive RA.

**Methods:**

In this population-based EIRA study, rheumatoid factor (RF), ACPA, smoking, SE and PTPN22*620W status was determined in incident RA cases and matched controls. MBL-high (*n *= 1330) and MBL-low (*n *= 1257) genotypes predicting MBL levels were constructed from four promoter and exon-1 polymorphisms in the *MBL2 *gene. Odds ratios with 95% confidence interval (OR, 95% CI) were calculated by logistic regression. In extended families (*n *= 316), previously reported data were re-analyzed, considering RF and smoking.

**Results:**

MBL-high genotypes tended to be associated with RF-negative (OR = 1.20, 95% CI 0.96-1.51) but not RF-positive (OR = 1.00, 95% CI 0.83-1.20) RA. Results divided by ACPA status did not differ. When stratified for smoking, MBL-high genotype was strongly associated with RF-negative RA in never smokers (OR = 1.82, 95% CI 1.24-2.69) but not in ever smokers (OR = 0.96, 95% CI 0.73-1.30). In never smokers, the association was observed in both the RF-negative/ACPA-negative (OR = 1.67, 95% CI 1.10-2.55) and RF-negative/ACPA-positive subgroups (OR = 3.07, 95% CI 1.37-6.89), and remained on an SE/PTPN22*620W negative background. In the extended families, the reported association between high MBL and RA was in fact confined to never smokers.

**Conclusions:**

High MBL may predispose to RF-negative RA but only in individuals who have never smoked. This illustrates the importance of phenotypic subgrouping in genetic studies.

## Introduction

In recent years, it has become evident that the subsets of rheumatoid arthritis (RA) that are autoantibody positive and negative, that is have rheumatoid factor (RF) or anti-citrullinated peptide antibody (ACPA) or both, not only differ clinically but also have distinct genetic and environmental risk profiles [1]. Thus, the risk associated with the strongest known environmental (smoking) and genetic (HLA-DRB1 shared epitope, or SE) susceptibility factors for RA seems to be restricted mainly to autoantibody-positive disease [2-4]. This also applies to several other risk alleles, including PTPN22*620W [5], each with only a modest effect on RA risk, whereas reports for the autoantibody-negative RA subset are sparse [6].

The *MBL2 *gene is one of several candidate genes, which have not yielded consistent risk association with RA. The *MBL2 *gene codes for the mannan-binding lectin (MBL) protein, which is part of innate immune defenses and is present in serum as well as in synovial fluid [7]. MBL is a soluble pattern recognition receptor that binds to sugar structures on microorganisms and modified self structures, including dying host cells (apoptotic/necrotic), immunoglobulins (agalactosylated IgG and certain forms of IgM and IgA), and immune complexes. Thus, MBL can bind potential arthritogenic agents and, after activation of the complement system, might induce inflammation within the joint [8,9]. Common variant alleles situated in both promoter and structural regions of the *MBL2 *gene influence the stability, function, and serum levels of the MBL protein [9], which can vary 10,000-fold between individuals but are stable for each individual over time [10]. These variants can be grouped together into MBL-high and MBL-low genotypes, which are known to be associated with MBL levels above and below the median population level (approximately 1,000 μg/L), respectively [11].

In a study on extended RA families, we previously found higher MBL levels in RA patients than in their first-degree relatives and in unrelated controls [12]. The RA patients also had increased frequency of MBL-high genotypes in one case-control study [13], whereas other studies have reported no association [14-20] or the opposite association [21-23].

Taken together, variants in the *MBL2 *gene and its protein product can be functionally relevant in RA pathogenesis, but previous inconsistent findings need to be reconsidered in light of the known etiological heterogeneity of this disease. Thus, we have investigated the impact of genetic variants of MBL on RA risk by using information from a large population-based case-control study of incident RA (Epidemiological Investigation of Rheumatoid Arthritis, or EIRA), and this enabled us to dissect this criteria-based syndrome into subgroups on the basis of autoantibody status and environmental (smoking) and genetic (SE and *PTPN22*) risk factors that are known to be associated mainly with the autoantibody-positive form. We found that the MBL-high genotype was associated with RF-negative RA but only in individuals who had never smoked. Similar findings were observed in the extended RA families [12], in whom the reported association between high MBL levels and RA was, in fact, confined to never smokers.

## Materials and methods

### Study group: The Epidemiological Investigation of Rheumatoid Arthritis

The study is a population-based case-control study that was initiated in 1996 and that encompasses incident cases of early RA from a geographically defined area in Sweden. For each case, a control subject was randomly selected from the Swedish national population registry, matched for age, sex, and residential area. In this study, we investigated 1,786 RA cases and 1,029 controls which were included in EIRA from 1996 to 2004 and had available DNA (88% of all participant cases and matched controls). All cases fulfilled the American College of Rheumatology 1987 criteria for the classification of RA. All participants gave informed consent and answered a questionnaire that included detailed information on environmental exposures. The cases and controls were classified according to their smoking habits into never or ever smokers (not available for 3% of the participants). The study was approved by the ethical review board of the Karolinska Institute.

### Replication study: extended Icelandic rheumatoid arthritis families

A replication study was performed in 74 extended Icelandic RA families, which have been described in detail in a previous report [12]. From the 210 RA patients and 406 first-degree relatives in the families, information about smoking habits was available for 53%. RF had been measured in all participants by using standard procedures, as previously described [24].

### Definition of variables

RF status was determined by using standard procedures and ACPAs by standard ELISA (Immunoscan-RA Mark2 ELISA test; Euro-Diagnostica, Malmö, Sweden). RF status was missing for 9%, and ACPA status was not available for 6%. The methods for determining the HLA-DRB1 SE alleles and the PTPN22*R620W (1858C/T) polymorphism have been previously reported [3,4,25]. Carriage of SE and the PTPN22*620W could not be defined for 1.2% and 1.7%, respectively.

MBL status was defined on the basis of genotyping in EIRA and serum levels in the extended RA families. MBL serum levels were measured by a sandwich ELISA system as previously described [19]. In EIRA, four single-nucleotide polymorphisms in the *MBL2 *gene were genotyped with two different methods. One regulatory *MBL2 *promoter polymorphism, influencing the production of MBL (rs7096206 in position -221, C/G, which is often referred to as minor allele X versus major allele Y), was genotyped by TaqMan allelic discrimination assay on a 384-well plate in accordance with recommendations of the manufacturer (Applied Biosystems, Foster City, CA, USA) (missing for 3.8%). By means of the pyrosequencing platform, a modified method from Roos and colleagues [26] was used on a 96-well plate (Supplementary Text 1). Three structural polymorphisms within exon-1, which interrupt the polymerization of the protein, were analyzed: rs5030737 in codon 52 (C/T or minor allele called D versus A), rs1800450 (codon 54, A/G or minor allele B versus A), and rs1800451 (codon 57, A/G or minor allele C versus A). This method is preferable to TaqMan for the exon-1 polymorphisms as they are so close to each other.

### Construction of a functional mannan-binding lectin genotype

To construct a functionally relevant genotype, the three minor alleles within exon-1 are pooled and referred to as 0 as opposed to A when not carrying any of these three minor alleles. The minor allele of the promoter polymorphism (referred to as X as opposed to the high-level-producing Y allele) always exists with major alleles (A) of all the exon-1 polymorphisms [9]. Therefore, only the following haplotypes are observed; XA, YA, YB, YC, or YD. YB, YC, and YD are pooled together and referred to as 0, as they result in similar MBL serum levels and only one can be present. Four individuals (0.1%) deviated from these known haplotypes (that is, genotype showed X together with B, C, or D) and were excluded. All detected genotypes were in Hardy-Weinberg equilibrium. A full composite functional MBL genotype was available for 2,586 of the 2,815 participants (YA/YA, YA/XA, XA/XA, 0/YA, 0/XA, or 0/0).

### Statistical analysis

Multivariate logistic regression was used to calculate odds ratios (ORs) with 95% confidence intervals (CIs) of carrying the MBL-high as compared with the MBL-low genotype, adjusted for age, sex, and residence of cases and controls. Additional analyses were performed by adding SE, PTPN22*620W, and smoking to the model. The Mann-Whitney test was used to compare continuous MBL levels between groups as they are not normally distributed. Statistical analyses were performed with SAS 9.1 software (SAS Institute Inc., Cary, NC, USA).

## Results

### Baseline characteristics

The EIRA study represents a typical early RA cohort. Seventy percent of the patients were female, the median age was 54 years (16 to 82), 66% were RF-positive, and 61% were ACPA-positive. Seventy percent of the cases and 65% of the age-, gender-, and geographic-location-matched controls had ever smoked.

Allele frequencies of the four polymorphisms in the *MBL2 *gene were in accordance with those reported from other Caucasian populations (Table 1). Variants that are known to be associated with MBL serum levels above and below the median population level (approximately 1,000 μg/L) were grouped together in functionally meaningful genotypes (see Materials and methods), and these were the basis for all analyses shown. MBL-high genotype (*n *= 1,330) refers to those with a major allele of all exon-1 polymorphisms, referred to as A/A, excluding homozygosity for the low-producing minor promoter allele (referred to as XA/XA). In the MBL-low genotype (*n *= 1,256), those genotypes that are associated with intermediate or deficient levels of MBL are pooled, including those with homo- or heterozygosity for the minor alleles of the exon-1 polymorphisms (0/0), referred to as 0/0, 0/YA and 0/XA, as well as those who are homozygous for the minor promoter allele (XA/XA).

**Table 1:** Allele frequencies of the whole EIRA study group

Position	**Genotype**^**a**^	Cases	Controls
Promoter/-221 nt (rs 7096206)			
	CC (YY)	1,130	609
	CG (XY)	542	310
	GG (XX)	65	51
	Missing	49	59
Exon 1			
Codon 52/223 nt (rs 5030737)			
	CC (AA)	1,420	826
	CT (AD)	282	148
	TT (DD)	14	10
	Missing	70	45
Codon 54/230 nt (rs 1800450)			
	GG (AA)	1,253	701
	AG (AB)	422	257
	AA (BB)	41	26
	Missing	70	45
Codon 57/239 nt (rs 1800451)			
	GG (AA)	1,643	955
	AG (AC)	71	29
	AA (CC)	2	0
	Missing	70	45
Exon 1 haplotype			
Wild-type	A/A	947	544
Heterozygous	0/A	657	373
Homozygous	0/0	112	67
	Missing	70	45
Functional MBL genotype^b^			
High	YA/YA+YA/XA	863	467
Low	YA/0, 0/0, XA/0, XA/XA	803	453
	Missing	120	109

^a^The nomenclature of the mannan-binding lectin (MBL) literature is shown within parentheses. ^b^0 refers to carrying any one of the exon 1 minor alleles (B, C, or D) and A is the major allele. The promoter minor allele X is in linkage equilibrium with the A major allele of exon 1. 0/0 refers to DD, BB, CC, or compound heterozygotes: BD, BC, or CD. EIRA, Epidemiological Investigation of Rheumatoid Arthritis.

### Does high mannan-binding lectin predispose to rheumatoid arthritis as a whole?

In the study group as a whole, no association was observed between MBL-high, as compared with MBL-low genotype, and RA (OR 1.03, 95% CI 0.87 to 1.21). Nor was any association between MBL-high genotype and RA observed when the association estimate was adjusted for three established risk factors for RA (smoking, SE, and PTPN22*620W) in the multivariate model (adjusted OR 0.99, 95% CI 0.83 to 1.18), but as expected, these risk factors were significantly associated with RA (ever smoking: adjusted OR 1.39, 95% CI 1.17 to 1.67; SE: adjusted OR 2.76, 95% CI 2.32 to 3.28; and PTPN22*620W: adjusted OR 1.47, 95% CI 1.21 to 1.79).

### Stratification by serological status

Next, to evaluate whether the MBL-high genotype might be a risk factor for a certain subgroup of the criteria-based syndrome (Table 2), the study group was stratified according to RF and ACPA status. Then, a non-significant trend association was observed for RF-negative RA (OR 1.20, 95% CI 0.96 to 1.51), whereas no association was observed for RF-positive RA (OR 1.00, 95% CI 0.83 to 1.20). No significant associations were observed when stratified for ACPA status alone (Table 2), but interestingly, the MBL-high genotype tended to be associated with the RF-negative/ACPA-positive subgroup of RA (OR 1.54, 95% CI 0.99 to 2.38) rather than the RF-negative/ACPA-negative subgroup (OR 1.14, 95% CI 0.89 to 1.47).

**Table 2:** Rheumatoid arthritis risk associated with MBL-high genotype in the whole EIRA study group and stratified for serology and smoking status

	Whole group		Never smokers	Ever smokers
	
	Number of cases/controls	**OR (95% CI)**^**a**^	**OR (95% CI)**^**a**^	**OR (95% CI)**^**a**^
Whole group	1,666/920	1.03 (0.87-1.21)	1.39 (1.04-1.85)	0.83 (0.67-1.04)
Stratification by serological status				
Seronegative				
RF-	476/920	1.20 (0.96-1.51)	1.82 (1.24-2.69)	0.96 (0.73-1.28)
ACPA-	585/920	1.03 (0.83-1.27)	1.42 (0.98-2.05)	0.77 (0.58-1.04)
RF-/ACPA-	361/920	1.14 (0.89-1.47)	1.67 (1.10-2.55)	0.80 (0.56-1.13)
RF-/ACPA+^b^	105/920	1.54 (0.99-2.38)	3.07 (1.37-6.89)	1.06 (0.57-1.99)
Seropositive				
RF+	925/920	1.00 (0.83-1.20)	1.26 (0.89-1.78)	0.86 (0.68-1.10)
ACPA+	916/920	1.08 (0.89-1.30)	1.55 (1.08-2.23)	0.88 (0.69-1.13)
RF+/ACPA-^c^	172/920	0.83 (0.59-1.17)	0.96 (0.53-1.74)	0.74 (0.47-1.17)
RF+/ACPA+	744/920	1.06 (0.87-1.29)	1.42 (0.97-2.10)	0.90 (0.70-1.16)

^a^Risk was calculated as odds ratios (ORs) with 95% confidence intervals (CIs) adjusted for age, sex, and geographic location by using logistic regression. ^b^In the ACPA+ group, 20% of never smokers and 10% of smokers were RF-: OR 2.07 (1.36 to 3.14), *P *= 0.0005. ^c^In the RF+ group, 24% of never smokers and 17% of smokers were ACPA-: OR 1.58 (1.11 to 2.25), *P *= 0.01. ACPA, anti-citrullinated protein antibody; EIRA, Epidemiological Investigation of Rheumatoid Arthritis; MBL, mannan-binding lectin; RF, rheumatoid factor.

### Stratification by smoking status

When the same exercise was performed after stratification by smoking status, the trend association observed between MBL-high genotype and RF-negative RA turned out to be confined to never smokers (OR 1.82, 95% CI 1.24 to 2.69) whereas no association was observed in ever smokers (OR 0.96, 95% CI 0.73 to 1.28). The association in never smokers was significant for both the RF-negative/ACPA-positive subgroup of RA (OR 3.07, 95% CI 1.37 to 6.89) and the RF-negative/ACPA-negative subgroup (OR 1.67, 95% CI 1.10 to 2.55). This was also significant for the whole ACPA-positive subgroup on a never-smoking background (OR 1.55, 95% CI 1.08 to 2.23), and a trend was observed for ACPA-negative RA (OR 1.42, 95% CI 0.98 to 2.05). Subgrouping of ever smokers by serological status yielded no significant associations between MBL-high genotype and RA (Table 2).

In fact, the association between the MBL-high genotype and RA was significant for the never-smoking group as a whole (Table 2), irrespectively of serological status (OR 1.39, 95% CI 1.04 to 1.85). From a more functional angle, never-smoking RA patients carrying the MBL-high genotype were less likely to be RF-positive (52%) as compared with patients carrying the MBL-low genotype (63%, OR 0.65, 95% CI 0.44 to 0.97), but no difference was observed in ever smokers (69% versus 70%). The proportion of ACPA-positive patients was similar in never-smoking RA patients carrying the MBL-high and MBL-low genotype (52% and 53%).

### Stratification by genetic risk factors

**Figure 1:**
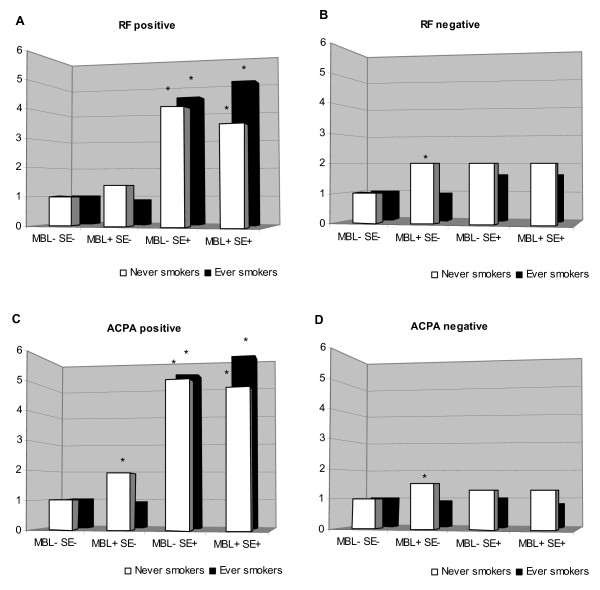
**Risk of developing rheumatoid arthritis in subjects exposed to different combinations of cigarette smoking status (never or ever smoker), MBL-high genotype, and the 'shared epitope'**. Subjects were stratified by the presence of rheumatoid factor (a,b) or anti-citrullinated protein antibodies (c,d). Risk is calculated as odds ratios by using logistic regression adjusted for age, sex, and geographic location. *Significant 95% confidence interval. ACPA, anti-citrullinated peptide antibody; MBL, mannan-binding lectin; RF, rheumatoid factor; SE, shared epitope.

**Figure 2:**
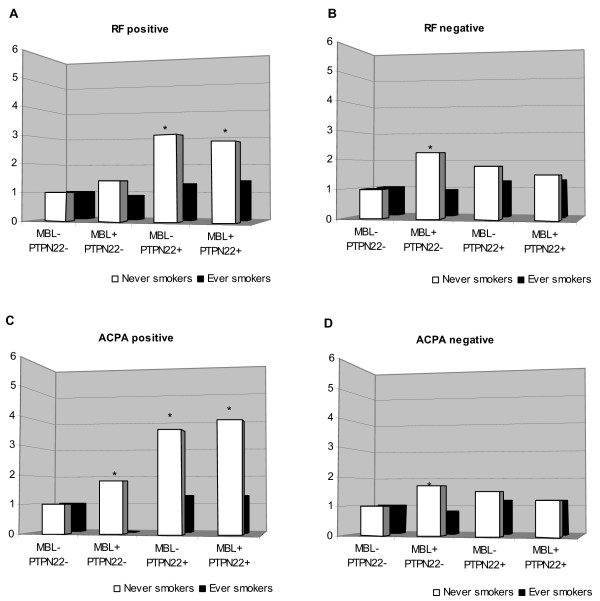
Risk of developing rheumatoid arthritis in subjects exposed to different combinations of cigarette smoking status (never or ever smoker), MBL-high genotype, and the PTPN22*620W risk allele. Subjects were stratified by the presence of rheumatoid factor (a,b) or anti-citrullinated protein antibodies (c,d). Risk is calculated as odds ratios by using logistic regression adjusted for age, sex, and geographic location. *Significant 95% confidence interval. ACPA, anti-citrullinated peptide antibody; MBL, mannan-binding lectin; RF, rheumatoid factor.

Then, we wanted to see, in the context of smoking status, whether there was an interaction between the MBL-high genotype and SE, the main genetic risk factor identified for RA. In Figure 1, the subgroups of autoantibody-positive and -negative disease are shown separately: RF-positive versus -negative (Figure 1a,b) and ACPA-positive versus -negative (Figure 1c,d), respectively. Never smokers not carrying the SE and the MBL-high genotype served as the referent group. As previously reported, carrying the SE was a strong risk factor for RF-positive but not for RF-negative RA. However, the MBL-high genotype was associated with a double risk of RF-negative RA in never smokers, but this was significant on an SE-negative background only. An overall similar pattern was observed in the ACPA-positive (Figure 1c) and ACPA-negative (Figure 1d) subgroups, although the MBL-high genotype was significantly associated with both subgroups on an SE-negative background in never smokers. A similar pattern was observed when stratified for the PTPN22*620W risk allele instead of SE (Figure 2).

### Additional evidence from a family study

As we have previously reported higher MBL levels in RA patients than in their first-degree relatives in Icelandic families [12], we went back to the families and re-analyzed the data for those 53% who had available information about smoking (106 RA patients and 210 first-degree relatives). Patients with or without information about smoking habits did not differ with respect to MBL levels (*P *= 0.5), age (*P *= 0.5), sex (*P *= 0.2), or RF positivity (*P *= 0.2). Furthermore, a similar difference was observed in MBL levels between RA patients and first-degree relatives who did not have information about smoking status (1,605 versus 989 μg/L; *P *= 0.11) as in those who did (1,573 versus 1,202 μg/L; *P *= 0.03).

**Figure 3:**
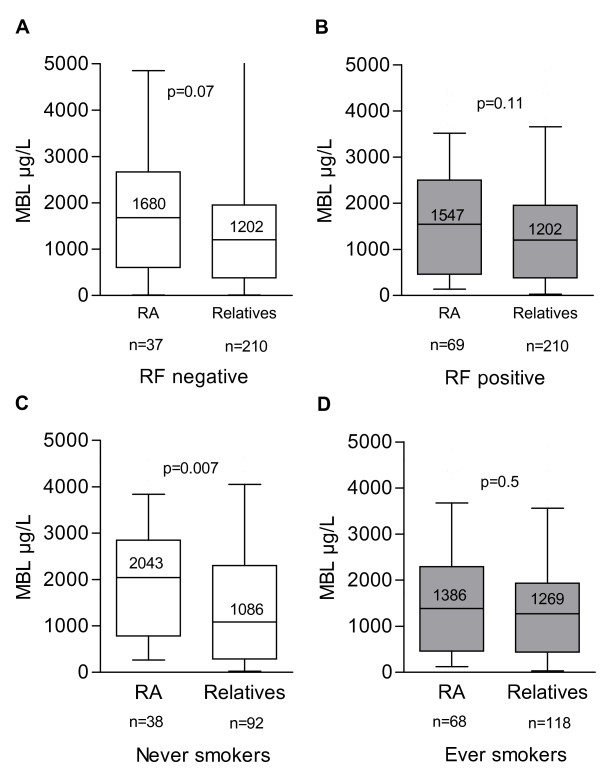
**MBL levels of rheumatoid arthritis patients from extended Icelandic families compared with their first-degree relatives**. Patients and relatives were stratified first according to their rheumatoid factor (RF) status into RF-negative (a) and RF-positive (b) and then according to their cigarette smoking status into never smokers (c) and ever smokers (d). The box plots show the median values and interquartiles, and the 10th percentiles of MBL concentrations are shown by the bars. MBL levels between two groups are compared by the Mann-Whitney rank sum test. MBL, mannan-binding lectin; RA, rheumatoid arthritis.

RF status was available for all patients, and 65% were RF-positive. Of these, 80% of the RA patients and 59% of their non-RA first-degree relatives were ever smokers (OR 2.72, 95% CI 1.57 to 4.70). As a MBL serum level of 1,000 μg/L is reported to distinguish fairly well between individuals with MBL-high and MBL-low genotypes, this cutoff was used for comparison as in the previous report. It was previously reported that patients with RA had higher MBL levels than their first-degree relatives. No significant association was observed when stratified for RF status into RF-positive disease (OR 1.25, 95% CI 0.72 to 2.19) and RF-negative disease (OR 1.40, 95% CI 0.67 to 2.89). When MBL levels were compared as a continuous variable, RF-negative RA patients tended to have higher MBL levels than relatives (Figure 3a; *P *= 0.07), but the findings were less significant when the larger group of RF-positive RA patients was compared with relatives (Figure 3b; *P *= 0.11).

However, when stratified for smoking status and analyzed with the 1,000 μg/L cutoff, the reported association turned out to be limited to the never smokers (OR 2.40, 95% CI 1.05 to 5.51), whereas no association was observed in ever smokers (OR 0.92, 95% CI 0.50 to 1.69). This was also observed when MBL levels were compared as a continuous variable, where never-smoking patients with RA had a median level two times higher than that of the relatives (Figure 3c; *P *= 0.007), whereas no difference was observed in ever smokers (Figure 3d; *P *= 0.5). When the never-smoking group was stratified further according to RF status, the MBL levels were also significantly higher in RF-negative RA patients than in the first-degree relatives (2,068 versus 1,086 μg/L; *P *= 0.036). Thus, these findings are similar to those in the EIRA study.

## Discussion

Our results indicate that functionally important genetic variations of the *MBL2 *gene, or high MBL levels, are associated with RF-negative RA, but only in individuals who have never smoked. These findings were detected in the EIRA case-control study and confirmed in a separate independent family-based study, in which high MBL was previously found to be associated with RA as a whole. This highlights the importance of careful subgrouping of the criteria-based clinical syndrome of RA since risk associations that exist only in subgroups of patients may otherwise not be detected. Thus, carrying the MBL-high genotype seems to double the risk of RF-negative RA in never smokers, namely a subgroup in which the main established genetic risk factors (SE and the PTPN22*620W allele) do not play a significant role. Analyzing this subset further by taking away those carrying the SE or PTPN22*620W allele showed an even stronger association with the MBL-high genotype, indicating that the pathogenic mechanisms involving MBL are not dependent on these variants.

These findings may explain those in previous studies, in which no association was observed between high MBL levels or associated genotypes and the risk of RA [14-19]. However, previous findings in the extended RA families, in which RA patients had higher MBL levels than their first-degree relatives, turned out to be limited to the never smokers, particularly the RF-negative subgroup [12]. Whether or to what extent smoking status might explain previous contradictory findings remains to be elucidated.

Given these results, high MBL is unlikely to play a role in the etiology of RF-positive RA, in which smoking, SE, and PTPN22*620W are well-known environmental and genetic risk factors [3-5]. MBL may, on the other hand, have a role in the pathogenesis of RF-negative disease but only in the absence of smoking as an environmental trigger. This is particularly interesting as the recent genome-wide association studies have yielded sparse results for RF-negative RA [6]. Actually, the findings in previous publications indicated that RF positivity is more frequent in those with lower MBL levels [19,23], and similar non-significant findings have been observed for MBL-low genotypes [15,22], but no previous report has compared RF-positive and -negative patients with controls separately.

Among the strengths of this study is the large, population-based recruitment of early RA cases and carefully matched controls. The participation rate was high, and detailed information about smoking status and validated genetic risk factors was available. The findings were then replicated in another independent Caucasian population in an extensive Icelandic family-based study, in which patients were compared with their first-degree relatives. This should minimize the potential confounding effect of genetic heterogeneity and environmental factors. The first study was based on a genotype (MBL-high genotype) known from previous studies to predict a certain phenotype (MBL levels above the median population level), whereas the replication study was based on the phenotype itself, namely the serum levels of the MBL protein, thus illustrating the functional relevance of these findings. In the family-based study, information about smoking was available for only 53% of the participants. Nevertheless, findings similar to those of the EIRA study were observed in the family study; namely, the previously reported association was, in fact, confined to the never-smoking group. Thus, the findings indicate that smoking somehow hinders the function of MBL.

Possible speculation why association is more consistent in non-smokers may be based on the hypothesis that MBL is inactivated in smokers. In accordance with this hypothesis, 65% of RF-negative RA patients but only 1.6% of controls have been reported to have anti-MBL antibodies, and in a later study, the authors found MBL to be S-nitrosylated (SNO-MBL) in a majority of the RA patients [27,28]. These antibodies are likely to influence the major function of MBL: its ability to opsonize apoptotic debris and microorganisms and to activate the complement system. Additionally, synovial fluid from RA patients is able to induce S-nitrosylation (SNO) of MBL, and anti-SNO-MBL was shown to be higher in synovial fluid than in serum [27,28]. Thus, post-translational modification of MBL by SNO may induce autoantibody production, which in turn may hinder its function. As cigarette smoke is the strongest known exogenous nitrosylating agent in the body [29,30], it is plausible that smoking inhibits the MBL function through nitrosylation by itself without involvement of autoantibody, and therefore, the risk of RA associated with MBL-high genotypes is observed only in never smokers.

Although the association with the MBL-high genotype was confined mostly to the RF-negative subgroup, similar findings were observed within both the ACPA-positive and -negative subgroups. Therefore, we dissected this further into subgroups according to both RF and ACPA status and found the association in the RF-negative subgroup to be significant in both those with and those without ACPA. Although this study was not designed to elucidate pathogenic mechanisms, a plausible explanation for this may lie in the inherent difference between these two autoantibody markers. ACPA binds citrullinated structures and is quite specific for RA, whereas RF is an anti-antibody that builds immune complexes and is observed in a substantial proportion of patients with other inflammatory diseases and healthy controls. Smoking induces RF production in healthy individuals [31] but can also lead to citrullination of lung structures and thereby trigger ACPA production in RA patients carrying a vulnerable genetic background [32]. MBL can bind to antibodies, including agalactosylated IgG (which is increased in RA patients), IgA, certain IgM isoforms, and immune complexes [8,33-35]. This supports the notion that MBL-mediated clearance of antibodies and immune complexes might diminish the likelihood of RF production and thereby seropositivity. As a pattern recognition receptor that has multiple binding sites, MBL has presumably higher affinity to immune complexes than single-antibody particles. It has been reported that patients with high MBL levels are less likely to be RF-positive [19,23], a finding that we could confirm in the never-smoking group of the EIRA study, whereas no difference was observed among smokers. Given this finding and the current literature, we hypothezise that MBL mediates the clearance of circulating immune complexes (and perhaps RF) from the blood but that, within confined spaces like the joint, MBL may lead to complement-mediated inflammation after binding to immune complexes, resulting in the syndrome of RF-negative RA.

## Conclusions

In a population-based case-control study and also in an extended family study, we have found that the MBL-high genotype or high levels of its product, the MBL protein, are associated with RF-negative RA in those who have never smoked. This highlights the importance of careful subgrouping of the criteria-based clinical syndrome of RA, as risk associations that exist only in subgroups of patients may otherwise not be detected.

## Abbreviations

ACPA: anti-citrullinated peptide antibody; CI: confidence interval; EIRA: Epidemiological Investigation of Rheumatoid Arthritis; ELISA: enzyme-linked immunosorbent assay; HLA: human leucocyte antigen; Ig: immunoglobulin; MBL: mannan-binding lectin; OR: odds ratio; PCR: polymerase chain reaction; RA: rheumatoid arthritis; RF: rheumatoid factor; SE: shared epitope; SNO: S-nitrosylation.

## Competing interests

The authors declare that they have no competing interests.

## Authors' contributions

SS was involved in the study conception and design, performed measurement of MBL in serum and genotyping, the statistical analyses, interpretation of the results, and drafted the manuscript. LP supervised the genotyping of MBL, conceived the discovery study and was involved in the data acquisition, and was involved in the study design and interpretation of the findings. HV supervised the measurement of MBL in serum. BD helped perform the statistical analyses. LK and LA conceived the discovery study and were involved in the data acquisition, the study design and interpretation of the findings. KS and GG conceived the replication study and were involved in the data acquisition. All authors read and approved the final manuscript.
